# Supplementary material for: Auxin-mediated protein depletion for metabolic engineering in terpene-producing yeast
Source: Nat Commun. 2021 Feb 16;12:1051. doi: 10.1038/s41467-021-21313-1 (PMC7886869; doi:10.1038/s41467-021-21313-1)
Supplement: Supplementary file 1 — Supplementary information [file 41467_2021_21313_MOESM1_ESM.pdf]

Supplementary information for:

Auxin-mediated protein depletion for metabolic engineering in terpene-producing yeast

Zeyu Lu[1,2], Bingyin Peng[1,3*], Birgitta E. Ebert[1,3], Geoff Dumsday[4], Claudia E. Vickers[1,3,5*]

[1] Australian Institute for Bioengineering and Nanotechnology (AIBN), the University of Queensland, Brisbane, QLD 4072, Australia

[2] School of Chemistry and Molecular Biosciences (SCMB), the University of Queensland, Brisbane, QLD 4072, Australia

[3] CSIRO Future Science Platform in Synthetic Biology, Commonwealth Scientific and Industrial Research Organisation (CSIRO), Black Mountain, ACT 2601, Australia

[4] CSIRO Manufacturing, Clayton, VIC 3169. Australia

[5] ARC Centre of Excellence in Synthetic Biology, Queensland University of Technology, Brisbane, QLD 4000, Australia

* Corresponding authors

Claudia E. Vickers (claudia.vickers@csiro.au)

Bingyin Peng (b.peng@uq.edu.au)

Supplementary Table 1. Plasmids used in this work

Plasmid	Properties	Reference
pILGFPE9	Yeast integration plasmid; P_{URA3}>*KlURA3*>T_{AgTEF1}-P_{TEF1}>*yEGFP*>T_{URA3}	[1]
pJAID6A	Yeast integration plasmid; P_{URA3}>*KlURA3*>T_{AgTEF1}-P_{TEF1}>*yEGFP*>T_{PGK1}-P_{ACS2}>$OsTIR1^{opt}$>T_{URA3}	This work
pJAID5Z	Yeast integration plasmid; P_{URA3}>*KlURA3*>T_{AgTEF1}-P_{TEF1}>*AID**>*yEGFP*>T_{PGK1}-P_{ACS2}>$OsTIR1^{opt}$>T_{URA3}	This work
pJAID5VY	Yeast integration plasmid; P_{URA3}>*KlURA3*>T_{AgTEF1}-P_{TEF1}>*CUP1-AID**>*yEGFP*>T_{PGK1}-P_{ACS2}>$OsTIR1^{opt}$>T_{URA3}	This work
pJAID6JY	Yeast integration plasmid; P_{URA3}>*KlURA3*>T_{AgTEF1}-P_{TEF1}>*TRX1-AID**>*yEGFP*>T_{PGK1}-P_{ACS2}>$OsTIR1^{opt}$>T_{URA3}	This work
pJAID5WY	Yeast integration plasmid; P_{URA3}>*KlURA3*>T_{AgTEF1}-P_{TEF1}>*SOD1-AID**>*yEGFP*>T_{PGK1}-P_{ACS2}>$OsTIR1^{opt}$>T_{URA3}	This work
pJAID5XY	Yeast integration plasmid; P_{URA3}>*KlURA3*>T_{AgTEF1}-P_{TEF1}>*mRFP-AID**>*yEGFP*>T_{PGK1}-P_{ACS2}>$OsTIR1^{opt}$>T_{URA3}	This work
pJAID9L	Different from pJAID6A by having different restriction sites; Yeast integration plasmid; P_{URA3}>*KlURA3*>T_{AgTEF1}-P_{TEF1}>*yEGFP*>T_{PGK1}-P_{ACS2}>$OsTIR1^{opt}$>T_{URA3}	This work
pJAID8Z	Yeast integration plasmid; P_{URA3}>*KlURA3*>T_{AgTEF1}-P_{TEF1}>*yEGFP*>T_{PGK1}-P_{ACS2}>*SKP1*>$OsTIR1^{opt}$>T_{URA3}	This work
pJAID9L104	Yeast integration plasmid; P_{URA3}>*KlURA3*>T_{AgTEF1}-P_{TEF1}>*yEGFP*>*AID**>*CUP1*>T_{PGK1}-P_{ACS2}>$OsTIR1^{opt}$>T_{URA3}	This work
pJAID9L105	Yeast integration plasmid; P_{URA3}>*KlURA3*>T_{AgTEF1}-P_{TEF1}>*yEGFP*>*AID**>*CUP1*>*SV40NSL*>T_{PGK1}-P_{ACS2}>$OsTIR1^{opt}$>T_{URA3}	This work
pJAID8Z104	Yeast integration plasmid; P_{URA3}>*KlURA3*>T_{AgTEF1}-P_{TEF1}>*yEGFP*>*AID**>*CUP1*>T_{PGK1}-P_{ACS2}> *SKP1*>$OsTIR1^{opt}$>T_{URA3}	This work
pJAID8Z105	Yeast integration plasmid; P_{URA3}>*KlURA3*>T_{AgTEF1}-P_{TEF1}>*yEGFP*>*AID**>*CUP1*>*SV40NSL*>T_{PGK1}-P_{ACS2}>*SKP1*>$OsTIR1^{opt}$>T_{URA3}	This work
pILGFP5V1	Yeast integration plasmid; P_{URA3}>*KlURA3*>T_{AgTEF1}-P_{TEF1}>*CUP1-AID**>*yEGFP*>T_{PGK1}- T_{URA3}	This work
pILGFP104	Yeast integration plasmid; P_{URA3}>*KlURA3*>T_{AgTEF1}-P_{TEF1}>*yEGFP*>*AID**>*CUP1*>T_{PGK1}-T_{URA3}	This work
pILGFP105	Yeast integration plasmid; P_{URA3}>*KlURA3*>T_{AgTEF1}-P_{TEF1}>*yEGFP*>*AID**>*CUP1*>*SV40NSL*>T_{PGK1}- T_{URA3}	This work
pML105	Yeast/E. coli shuttle plasmid; *2μ URA3* P_{TDH3}>*Cas9*>T_{ADH1}-$T_{SUP4} < \mathit{Swa}\mathrm{I} < P_{SNR52}$	[2]
pISKP1-TIR1	Yeast integration plasmid; P_{URA3}>*KlURA3*>T_{AgTEF1}- T_{PGK1}-P_{ACS2}> *SKP1*>$OsTIR1^{opt}$>T_{URA3}	This work

pJT11	Yeast/E. coli shuttle plasmid; *2µ LEU2* T_{RPL3}>*ERG20*^{N127W}>P_{GAL1}-P_{GAL10}>-*Part*$_{ALD6}$-P_{GAL2}>*ClLS*>T_{RPL41B}	[3]
pJT11F	Yeast/E. coli shuttle plasmid; *2µ LEU2* T_{RPL3}>*ERG20*^{N127W}>P_{GAL1}-P_{GAL10}>*Y-FAST*>T_{ALD6}-P_{GAL2}>*ClLS*>T_{RPL41B}	This work
pILGFP3	Yeast integration plasmid; P_{URA3}>*KlURA3*>T_{AgTEF1}-*yEGFP*>T_{URA3}	[1]
pILGFP1D5	Yeast integration plasmid; P_{URA3}>*KlURA3*>T_{AgTEF1}-*yEGFP*>T_{PGK1}-T_{URA3}	This work
pILGFP1F5	Yeast integration plasmid; P_{URA3}>*KlURA3*>T_{AgTEF1}-P_{TEF1}>*(BamHI)yEGFP*>T_{PGK1}-T_{URA3}	This work
pILGFP1H5	Yeast integration plasmid; P_{URA3}>*KlURA3*>T_{AgTEF1}-P_{TEF1}>*yEGFP*>T_{PGK1}-T_{URA3}	This work
pILGFPY2A	Yeast integration plasmid; P_{URA3}>*KlURA3*>T_{AgTEF1}-P_{TEF1}>*Y-FAST-ErB1.2A-yEGFP*>T_{PGK1}-T_{URA3}	This work
pJT9R	Yeast/E. coli shuttle plasmid; *2µ LEU2* T_{RPL3}>*ERG20*>P_{GAL1}-P_{GAL2}>*AcNES1*>T_{RPL41B}	[4]
pJT9RFR	Yeast/E. coli shuttle plasmid; *2µ LEU2* T_{RPL3}>*ERG20*>P_{GAL1}-P_{GAL2}>*Y-FAST*>*ErB1.2A-AcNES1*>T_{RPL41B}	This work
pIR3DH8	Yeast integration plasmid; *gal80Arm1*-P_{AgTEF1}-*KlURA3*-T_{AgTEF1}-*gal80Arm2*	This work
pIR3DH8K	Yeast integration plasmid; *gal80Arm1*-P_{TPI1}-*KanMX4*-*gal80Arm2*	This work

Supplementary Table 2. *Saccharomyces cerevisiae* strains used in this work

Strain	Genotype	Reference
CEN.PK2-1C	MATa ura3-52 trp1-289 leu2-3,112 his3Δ 1	[5]
CEN.PK113-5D	MATa ura3-52	[5]
ILHA series strains		
GH4	CEN.PK113-5D derivative; *ura3(1, 704)::KlURA3>*T_{AgTEF1}	[1]
G6A	CEN.PK113-5D derivative; *ura3(1, 704):: KlURA3>*T_{AgTEF1}-P_{TEF1}*>yEGFP>*T_{PGK1}-P_{ACS2}*>OsTIR1*^{opt}<br>(Figure 1, *yEGFP* + *TIR1*)	This work
G5Z	CEN.PK113-5D derivative; *ura3(1, 704):: KlURA3>*T_{AgTEF1}-P_{TEF1}*>AID*>yEGFP>*T_{PGK1}-P_{ACS2}*>OsTIR1*^{opt}<br>(Figure 1, *AID*-yEGFP* + *TIR1*)	This work
G5V	CEN.PK113-5D derivative; *ura3(1, 704):: KlURA3>*T_{AgTEF1}-P_{TEF1}*>CUP1-AID*>yEGFP>*T_{PGK1}-P_{ACS2}*>OsTIR1*^{opt}<br>(Figure 1, *CUP1-AID*-yEGFP* + *TIR1*)	This work
G6J	CEN.PK113-5D derivative; *ura3(1, 704):: KlURA3>*T_{AgTEF1}-P_{TEF1}*>TRX1-AID*>yEGFP>*T_{PGK1}-P_{ACS2}*>OsTIR1*^{opt}<br>(Figure 1, *TRX1-AID*-yEGFP* + *TIR1*)	This work
G5W	CEN.PK113-5D derivative; *ura3(1, 704):: KlURA3>*T_{AgTEF1}-P_{TEF1}*>SOD1-AID*>yEGFP>*T_{PGK1}-P_{ACS2}*>OsTIR1*^{opt}<br>(Figure 1, *SOD1-AID*-yEGFP* + *TIR1*)	This work
G5X	CEN.PK113-5D derivative; *ura3(1, 704):: KlURA3>*T_{AgTEF1}-P_{TEF1}*>mRFP-AID*>yEGFP>*T_{PGK1}-P_{ACS2}*>OsTIR1*^{opt}<br>(Figure 1, *SOD1-AID*-yEGFP* + *TIR1*)	This work
G5V1	CEN.PK113-5D derivative; *ura3(1, 704):: KlURA3>*T_{AgTEF1}-P_{TEF1}*>CUP1-AID*>yEGFP>*T_{PGK1}<br>(Figure 1, *CUP1-AID*-yEGFP W/O TIR1*)	This work
G9L104	CEN.PK113-5D derivative; *ura3(1, 704):: KlURA3>*T_{AgTEF1}-P_{TEF1}*>yEGFP>AID*>CUP1>*T_{PGK1}-P_{ACS2}*>OsTIR1*^{opt}<br>(Figure 1, *yEGFP-AID*-CUP1* + *TIR1*)	This work
G8Z104	CEN.PK113-5D derivative; *ura3(1, 704):: KlURA3>*T_{AgTEF1}-P_{TEF1}*>yEGFP>AID*>CUP1>*T_{PGK1}-P_{ACS2}*> SKP1>OsTIR1*^{opt}<br>(Figure 1, *yEGFP-AID*-CUP1* + *SKP1-TIR1*)	This work
G104	CEN.PK113-5D derivative; *ura3(1, 704):: KlURA3>*T_{AgTEF1}-P_{TEF1}*>yEGFP>AID*>CUP1>*T_{PGK1}<br>(Figure 1, *yEGFP-AID*-CUP1 W/O TIR1*)	This work
G9L105	CEN.PK113-5D derivative; *ura3(1, 704):: KlURA3>*T_{AgTEF1}-P_{TEF1}*>yEGFP>AID*>CUP1>SV40NSL>*T_{PGK1}-P_{ACS2}*>OsTIR1*^{opt}	This work

	(Figure 1, yEGFP-AID*-CUP1-NLS + TIR1)	
G8Z105	CEN.PK113-5D derivative; ura3(1, 704):: KlURA3>T_{AgTEF1}-P_{TEF1}>yEGFP>AID*>CUP1>SV40NSL>T_{PGK1}-P_{ACS2}>SKP1>OsTIR1^{opt}<br>(Figure 1, yEGFP-AID*-CUP1-NLS + SKP1-TIR1)	This work
G105	CEN.PK113-5D derivative; ura3(1, 704):: KlURA3>T_{AgTEF1}-P_{TEF1}>yEGFP>AID*>CUP1>SV40NSL>T_{PGK1}<br>(Figure 1, yEGFP-AID*-CUP1 W/O TIR1)	This work
G1H5	CEN.PK113-5D derivative; ura3(1, 704)::KlURA3-P_{TEF1}-yEGFP-T_{PGK1}	This work
Y2A-GFP	CEN.PK113-5D derivative; ura3(1, 704)::KlURA3-P_{TEF1}-Y-FAST-2A-yEGFP-T_{PGK1}	This work
o401R	CEN.PK2-1C derivative;<br>HMG2^{K6R}(−152 ,−1)::HIS3-T_{EFM1}<EfmvaS<P_{GAL1}–P_{GAL10}>ACS2>T_{ACS2}–P_{GAL2}>EfmvaE>T_{EBS1}–P_{GAL7}<br>pdc5 (−31, 94)::P_{GAL2}>ERG12>T_{NAT5}–P_{TEF2}>ERG8>T_{IDP1}–T_{PRM9}<MVD1<P_{ADH2}–T_{RPL15A}<IDI1<P_{TEF1}-TRP1<br>ERG9(1333, 1335)::T_{URA3}- P_{GAL7}>MVD1>T_{PRM9}-P_{GAL2}>ERG12>T_{NAT5}-T_{IDP1}<ERG8<P_{GAL10}-P_{GAL1}>IDI1>T_{RPL15A}-loxP-ble-loxP	[3]
o401RS	o401R derivative; ura3(1, 704):: KlURA3–T_{PGK1}-P_{ACS2}>SKP1-OsTIR1	This work
o141R	o401R derivative;<br>ERG20(-32, 3)::CUP1-AID*<br>ura3(1, 704)::KlURA3–T_{PGK1}-P_{ACS2}>SKP1-OsTIR1	This work
o128R	o401R derivative;<br>HXK2(1459, 1452)::AID*CUP1<br>ura3(1, 704)::KlURA3–T_{PGK1}-P_{ACS2}>SKP1-OsTIR1	This work
o138R	o401R derivative;<br>ACC1(0, 0)::CUP1-AID*<br>ura3(1, 704)::KlURA3–T_{PGK1}-P_{ACS2}>SKP1-OsTIR1	This work
LIM401	o401RS derivative; [pJT11F] gal80::P_{AgTEF1}>KanMX4> T_{AgTEF1}	This work
LIM141	o141R derivative; [pJT11F] gal80::P_{AgTEF1}>KanMX4>T_{AgTEF1}	This work
NLD4019R	o401RS derivative; [pJT9R] gal80::P_{TPI1}>KanMX4	This work
NLD401	o401RS derivative; [pJT9RFR] gal80::P_{TPI1}>KanMX4	This work
NLD128-1	o138R derivative; [pJT9RFR] gal80::P_{TPI1}> KanMX4	This work
NLD138	o128R derivative; [pJT9RFR] gal80::P_{TPI1}> KanMX4	This work

Supplementary Table 3: List of primers and PCR fragments used in this work. P_{XXX} and T_{XXX} indicate promoter and terminator sequence of gene XXX, respectively; sequences *in italic and red* indicate sequences complimentary to the DNA template; restriction enzyme sites used in cloning are shown in bold; 20-mer CRISPR/Cas9 guider sequence is **underlined in bold and red**.

#	Overlap extension PCR fragment	PCR fragment	Primer name	Sequence (5' → 3')
1	T_{PGK1}-P_{TIP1}-$OzTIR1^{opt}$	T_{PGK1} from SGD	PPGE9PGK1ts	GATGAATTGTACAAAAGATCTTAAACTAGT*ATTGAATTGAATTGAAATCGATAG*
			PPGE9PGK1ta	GTTAAATATCCGTAATCTTTAAACAGCGGCCGC*AAATAATATCCTTCTCGAAAGC*
		P_{TIP1} from SGD	PPGE9TPI1ps	GCTTTCGAGAAGGATATTATTTGCGGCCGC*TGTTTAAAGATTACGGATATTTAAC*
			PPGE9TPI1pa	CTTCTTCTGGGAAATAGGTCATTTTGCTAGC*TTTTAGTTTATGTATGTGTTTTTTG*
		$OsTIR1^{opt}$, codon-optimised *TIR1* from *Oryza sativa*	PPGE9TIR1s	CAAAAAACACATACATAAACTAAAAGCTAGCAAA*ATGACCTATTTCCCAGAAGAAG*
			PPGE9TIR1a	CCTTCCCTTTGCAAATAGTCCT*TTACAAAATCTTTACGAAATTTGG*
2		P_{ACS2} from SGD	PADACS2ps	GCTTTCGAGAAGGATATTATTTGCGGCCGC*GATTGCAAGAATTGATCTCC*
			PADACS2pa	CTTCTGGGAAATAGGTCATTTTGCTAGAA*TTTATTATTGTATTGATTTACTTTCCTG*
3		*AID*, codon-optimised minimal auxin inducible degron from IAA15*	PADUAID*s1	CAATCTAATCTAAGTTTTAATTACAAACTCGAGAAAATG*CCAAAAGACCCAGCTAAACC*
			PADUAID*a	GTGAATAATTCTTCACCTTTAGAGGAT*CCCATCTTAACAAAGGCAGCAGCTTCTGG*
4	*CUP1-AID**	*CUP1* from SGD	PADUCUP1s	CAATCTAATCTAAGTTTTAATTACAAACTCGAGAAA*ATGTTCAGCGAATTAATTAACTTC*
			PADUCUP1a	CTTGGCTGGAGGTTTAGCTGGGTCTTTTGG*TTTCCCAGAGCAGCATGACT*
		*AID** (1)	PADUAID*s	*CCAAAAGACCCAGCTAAACCTCCAGCCAAG*
			PADUAID*a	As above
5	*SOD1-AID**	*SOD1* from SGD	PADUSOD1s	CAATCTAATCTAAGTTTTAATTACAAACTCGAGAAA*ATGGTTCAAGCAGTCGCAGT*
			PADUSOD1a	CTTGGCTGGAGGTTTAGCTGGGTCTTTTGG*GTTGGTTAGACCAATGACAC*
		*AID** (1)	As above	
6	*mRFP-AID**	*mRFP*	PADDRFPs	CAATCTAATCTAAGTTTTAATTACAAACTCGAGAAA*ATGGCTTCCTCCGAAGACGT*
			PADDRFPa	CTTGGCTGGAGGTTTAGCTGGGTCTTTTGG*AGCACCGGTGGAGTGACGAC*
		*AID** (1)	As above	
7	*TRX1-AID**	*TRX1*	PADUTRX1s	CAATCTAATCTAAGTTTTAATTACAAACTCGAGAAA*ATGGTTACTCAATTCAAAACTG*
			PADUTRX1a	CTTGGCTGGAGGTTTAGCTGGGTCTTTTGG*AGCATTAGCAGCAATGGCTTG*
		*AID** (1)	As above	

8		T_{PGK1}-P_{TIP1}-*OzTIR1*^{opt} (XhoI) from pJAID6A	PPGE9PGK1ts	As above
			PPGE9TIR1a1	CCTTCCCTTTGCAAATAGTCCTCTCGAG*TTACAAAATCTTTACGAAATTTGG*
9		*SKP1* from SGD	PAIDSKP1s1	CAGGAAAGTAAATCAATACAATAATAAAAGCTAGCAAA*ATGGTGACTTCTAATGTTGTC*
			PAIDSKP1adaa1	GGTCACCAGCACCAGCACCAGCACCCAAATCAAAGATACC*ACGGTCTTCAGCCCATTCAT*
			PAIDSKP1a1	TGTTCAACGACTTCTTCTGGGAAATAGGTCATAGAAGTAA*GGTCACCAGCACCAGCACCA*
10	*AID*-CUP1*	*AID* (2)* from pJAID5VY	PJRRCAIDs	GTATGGATGAATTGTACAAAAGATCT*CCAAAAGACCCAGCTAAACC*
			pJRRCAIDa	GATCTATCGATTTCAATTCAATTCAATACTAGTTTA*CTTAACAAAGGCAGCAGCTTC*
		CUP1 (1) from SGD	pJRRCUP1s	TCCTCCGGTGGTCCAGAAGCTGCTGCCTTTGTTAAG*ATGTTCAGCGAATTAATTAACTTC*
			pJRRCUP1a	GATCTATCGATTTCAATTCAATTCAATACTAGTTTA*TTTCCCAGAGCAGCATGACT*
11	*AID*-CUP1-SV40NSL*	*AID* (2) CUP1-SV40NSL*	As above	
			pJRRCUP1s	As above
			pJRRCUP1NLSada	AACCTTTCTTTTCTTCTTAGGTGGGTACA*TTTCCCAGAGCAGCATGACT*
			pJRRCUP1NLSa	GATCTATCGATTTCAATTCAATTCAATACTAGTTTA*AACCTTTCTTTTCTTCTTAGGTGG*
12		*Y-FAST* from a synthetic gene fragment with codon optimised	GA_YFAST_PJT_Fwd	GAATTTTTGAAAATTCAATATAAA*ATGGAACACGTTGCTTTCGG*
			GA_YFAST_PJT_Rev1	AAGCAAAGTATAGGCTTTATTA*AACTCTTTTAACGAAAACCCAGTAAGAG*
13		T_{PGK1} from SGD	PPGPGK1ts	GGATGAATTGTACAAAAGATCTTAA*ATTGAATTGAATTGAAATCGATAG*
			PPGPGK1ta	CCCTTTGCAAATAGTCCTACTAGT*AAATAATATCCTTCTCGAAAGC*
14		P_{TEF1} from SGD	PPGTEF1ps2	AAGGGTTGCTCGAGAAAGAGCTC*CAGAAAGCGACCACCCAAC*
			PPGTEF1pa2	CAGTGAATAATTCTTCACCTTTAGAGGATCC*CATTTTGTAATTAAAACTTAGATTAG*
15		P_{TEF1} from SGD	PPGTEF1ps2	As above
			PPGTEF1pa3	CAGTGAATAATTCTTCACCTTTAGACAT*TTTGTAATTAAAACTTAGATTAG*
16		*Y-FAST-2A* from a synthetic gene fragment with codon optimised	PPGYFASTs1	CTAATCTAAGTTTTAATTACAAA*ATGGAACACGTTGCTTTCGGTTC*
			PPGYFAST2Aa	TGAATAATTCTTCACCTTTAGA*TGGACCTGGGTTAAGTTCAAC*
17		*Y-FAST-2A* (2)	PNESYFASTs2	CATAATAAAAAAAATAATTCTTTCAGTGGATCCAAA*ATGGAACACGTTGCTTTCGGTTC*
			PNESYFASTa	CGATTTGCTCTCAATCCGCCATGGTCACTA*TTAAACTCTTTTAACGAAAACCCAG*

18	*gal80Arm1*-P_{AgTEF1}-*KlURA3*-T_{AgTEF1}-*gal80Arm2*	*gal80Arm1* from SGD	PGAL80uarms	GTAAAACGACGGCCAGTGAATTCGTTT*AAACGGCCGCCTCTGCCATG*
			PGAL80uarma	ATCACTATCTCTTAGCATCT*TTAAACAGAGACATCGTCCG*
		P_{AgTEF1}-*KlURA3*-T_{AgTEF1} from pUG72	PGAL80URA3s	CGGACGATGTCTCTGTTTAA*AGATGCTAAGAGATAGTGAT*
			PGAL80URA3a	CGAAGATCTCTTGTTGTAGTCCTCGAG*CTTTACAGTCCTGTCTTATTG*
		gal80Arm2 from SGD	PGAL80darms	CAATAAGACAGGACTGTAAAGCTCGAG*GACTACAACAAGAGATCTTCG*
			PGAL80darma	GATTACGCCAAGCTTGCATGCGTTT*AAACCATTGCATTTATCCTGG*
19		*ERG20* guider	PCRISPRs	TGAAAGTTGGTGCGCATGTTTCGGCGTTCGAAACTTCTCC*GCAGTGAAAGATAAATGATC*
			PAIDCRISPRERG20	GCAGTGAAAGATAAATGATC**AACAGGTATTGGACTGACAT**GTTTTAGAGCTAGAAATAGC
			PCRISPRa	ACTTTTTCAAGTTGATAACGGACTAGCCTTATTTTAACTT*GCTATTTCTAGCTCTAAAC*
20		*CUP1-AID** donor (*ERG20*), amplified from pJAID5VY	PAIDERG20AIDs	TTAACTTTAAAAACTCAACCAACAGGTATTGGACTGACAT*ATGTTCAGCGAATTAATTAAC*
			PAIDERG20AIDa	CGTTCAAGAATCTCTCTCTCCTAATTTCTTTTTCTGAAGC*CTTAACAAAGGCAGCAGCTT*
21		Verification of *ERG20* locus	PvERG20s	ATAGAGGAAGCAACGGCAGG
			PvERG20a	GTTCAATGAGTGGGCATACCAGTC
22		*HXK2* guider	PCRISPRs	As above
			PAIDCRISPRHXK2	GCAGTGAAAGATAAATGATC**TTAAGCACCGATGATACCAA**GTTTTAGAGCTAGAAATAGC
			PCRISPRa	As above
23		*AID*-CUP1* donor (*HXK2*), amplified from pJAID9L104	PAIDHXK2AIDs	AAGAATTGCTGAAGGTAAGTCCGTTGGTATCATCGGTGCT*CCAAAAGACCCAGCTAAACC*
			PAIDHXK2AIDa	ACCTTCTTGTTGTTCAAACTTAATTTACAAATTAAGTTTA*TTTCCCAGAGCAGCATGACT*
24		Verification of *HXK2* locus	PvHXK2s	TGCTGAAGATGGTTCCGGTGC
			PvHXK2a	TATCGTCACGAATAAATCCCGTG
25		*ACC1* guider	PCRISPRs	As above
			PAIDCRISPRACC1-1	GCAGTGAAAGATAAATGATC**GAATAAGCTTTCTTCGCTCA**GTTTTAGAGCTAGAAATAGC
			PCRISPRa	As above
26		*CUP1-AID** donor (*ACC1*), amplified from pJAID5VY	PAIDACC1AIDs-1	AACAGCGCAGAAAATTAGAAAAAATCAAGTTTCTACC*ATGTTCAGCGAATTAATTAACTT*
			PAIDACC1AIDa-1	TCTTCTGTGGAGAAGACTCGAATAAGCTTTCTTCGCTCAT*CTTAACAAAGGCAGCAGCTT*
27		Verification of *ACC1* locus	PvACC1s-1	TAATAACCGATTCGTCTTCTAGC
			PvACC1a-1	GAAGTTCTGTATGTCTTTCTGAG
28	P_{TPI1}-*KanMX4*	P_{TPI1} from SGD	GA_pURA3_pTPI1_F	CCAACTGCACAGAACAAAAACC*ATTTAAACTGTGAGGACCTTAATAC*

		pTPI1_R	CCAT*TTTTAGTTTATGTATGTGTTTTTTG*
	KanMX4 from pUG6	pTPI1_KANMX-F	CTACAAAAAACACATACATAAACTAAAA*ATGGGTAAGGAAAAGACTCACG*
		GA_KANR_GAL80_R	CCGAAGATCTCTTGTTGTAGTC*TTAGAAAAACTCATCGAGCATC*
29	KanMX4 pUG6	PdGAL80s3	CAGCGTATACAATCTCGATAGTTGGTTTCCCGTTCTTTC*CAGCTGAAGCTTCGTACGCTG*
		PdGAL80a3	CAATAGAAGTCTCAATTTTTGGACTGTATAAGGCAGTG*ATATAGGCCACTAGTGGATCTG*

Supplementary Table 4. Construction of the plasmids used in this work. Numbers refer to DNA fragments listed in Supplementary Table 3.

Plasmid	Construction process
pJAID6A	Fragment *T_{PGK1}-P_{TIP1}-OzTIR1^{opt}* (#1) was cloned into *Spe*I site of pILGFPE9 through Gibson assembly to generate pJAID3OR. *P_{ACS2}* (#2) was cloned into *Not*I/*Nhe*I sites of pAID3OR to replace *P_{TPI1}* and generate pAID6A.
pJAID5Z	Fragment *AID** (#3) was cloned into *Xho*I/*Bam*HI site of pJAID6A through Gibson assembly to generate pJAID5Z
pJAID5VY	Fragment *CUP1-AID** (#4) was cloned into *Xho*I/*Bam*HI sites of pJAID6A through Gibson assembly to generate pJAID5Z
pJAID5WY	Fragment *SOD1-AID** (#5) was cloned *Xho*I/*Bam*HI sites of pJAID6A through Gibson assembly to generate pJAID5WY
pJAID5XY	Fragment *mRFP-AID** (#6) was cloned *Xho*I/*Bam*HI sites of pJAID6A through Gibson assembly to generate pJAID5WY
pJAID6JY	Fragment *TRX1-AID** (#7) was cloned *Xho*I/*Bam*HI sites of pJAID6A through Gibson assembly to generate pJAID5JY
pJAID9L	Fragement *T_{PGK1}-P_{ACS2}-OzTIR1^{opt}* (*Xho*I; #8) was cloned into *Spe*I site of pILGFPE9 through Gibson assembly to generate pJAID9L
pJAID8Z	Fragment *SKP1* (#9) was cloned *Nhe*I site of pJAID9L through Gibson assembly to generate pJAID8Z
pJAID9L104	Fragment *AID*-CUP1* (#10) into *Spe*I site of pJAID9L through Giboson assembly to generate pJAID9L104
pJAID9L105	Fragment *AID*-CUP1-SV40NLS* (#11) into *Spe*I site of pJAID9L through Giboson assembly to generate pJAID9L104
pJAID8Z104	Fragment *AID*-CUP1* (#10) into *Spe*I site of pJAID8Z through Giboson assembly to generate pJAID8Z104
pJAID8Z105	Fragment *AID*-CUP1-SV40NLS* (#11) into *Spe*I site of pJAID8Z through Giboson assembly to generate pJAID8Z104
pILGFP5V1	*Not*I/*Psi*I-digested pJAID5VY was treated with mung bean nuclease and self-ligated to generate pILGFP5V1
pILGFP104	*Not*I/*Psi*I-digested pJAID9L104 was treated with mung bean nuclease and self-ligated to generate pILGFP104
pILGFP105	*Not*I/*Psi*I-digested pJAID9L105 was treated with mung bean nuclease and self-ligated to generate pILGFP105
pISKP1-TIR1	*Xba*I/*Spe*I-digested pJAID8Z was self-ligated to generate pISKP1-TIR1
pJT11F	Fragment *Y-FAST* (#12) was cloned into *Spe*I site of pJT11 through Gibson assembly to generate pJT11F
pILGFP1D5	Fragment *T_{PGK1}* (#13) was cloned into *Spe*I site of pILGFP3 through Gibson assembly to generate to generate pILGFP1D5
pILGFP1F5	Fragment *P_{TEF1}* (#14) was cloned into *Bam*HI site of pILGFP1D5 through Gibson assembly to generate pILGFP1F5
pILGFP1H5	Fragment *P_{TEF1}* (#15) was cloned into *Bam*HI site of pILGFP1D5 through Gibson assembly to generate pILGFP1H5

pILGFPY2A	Fragment Y-FAST-2A (#16) was cloned into *Bam*HI site of pILGFP1F5 through Gibson assembly to generate pILGFPY2A
pJT9RFR	Fragment Y-FAST-2A (#17) was cloned into *Bam*HI site of pJT9R through Gibson assembly to generate pJT9FR.
pIR3DH8	Fragment *gal80Arm1*-P_{AgTEF1}-*KlURA3*-T_{AgTEF1}-*gal80Arm2* (#18) was cloned into EcoRI/SphI sites of pUC19 through Gibson assembly to generate pIR3DH8.
pIR3DH8K	Fragment P_{TPI1}- *KanMX4* (#28) was cloned into PstI/XhoI sites of pIR3DH8 through Gibson assembly to generate pIR3DH8K.

Supplementary Table 5. Construction of the ILHA series strains used in this work. Plasmids refer to Supplementary Table 1. DNA fragments refer to Supplementary Table 3.

Strain	Construction process
G6A	Plasmid pJAID6A digested with *Swa*I was transformed into CEN.PK113-5D to generate strain G6A, and:
G5Z	pJAID5Z to generate strain G5Z,
G5V	pJAID5VY to generate strain G5V,
G6J	pJAID6JY to generate strain G6J,
G5W	pJAID5WY to generate strain G5W,
G5X	pJAID5XY to generate strain G5X,
G5V1	pILGFP5V1 to generate strain G5V1,
G9L104	pJAID9L104 to generate strain G9L104,
G8Z104	pJAID8Z104 to generate strain G8Z104,
G104	pILGFP104 to generate strain G104,
G9L105	pILGFP9L105 to generate strain G9L105,
G8Z105	pJAID8Z105 to generate strain 8Z105,
G105	pILGFP105 to generate strain G105,
G1H5	pILGFP1H5 to generate strain G1H5,
Y2A-GFP	pILGFPY2A to generate strain Y2A-GFP.
o401RS	Plasmid pISKP1-TIR1 digested with SwaI was transformed into strain o401R to generate strain o401RS
o141R	Step 1: SwaI-digested pML104, *ERG20* guider (#19), *CUP1-AID** donor (*ERG20*; #20) were co-transformed into strain o401R;<br>Step 2: *URA3* plasmid (derived from pML104) was removed from Step 1 resulting strain;<br>Step 3: Plasmid pISKP1-TIR1 digested with SwaI was transformed into Step 2 resulting strain to generate strain o141R.
o128R	Step 1: SwaI-digested pML104, *HXK2* guider (#22), *AID8-CUP1* donor (*HXK2*; #23) were co-transformed into strain o401R;<br>Step 2: *URA3* plasmid (derived from pML104) was removed from Step 1 resulting strain;<br>Step 3: Plasmid pISKP1-TIR1 digested with SwaI was transformed into Step 2 resulting strain to generate strain o141R.
o138R	Step 1: SwaI-digested pML104, *ACC1* guider (#25), *CUP1-AID** donor (*ACC1*; #26) were co-transformed into strain o401R;<br>Step 2: *URA3* plasmid (derived from pML104) was removed from Step 1 resulting strain;<br>Step 3: Plasmid pISKP1-TIR1 digested with SwaI was transformed into Step 2 resulting strain to generate strain o141R.
LIM401 / LIM141	Step 1: Plasmid pJT11F was transformed into o401RS / o141R;<br>Step 2: Fragment *KanMX4* (#29) was transformed into Step 1 resulting strain to generate LIM401 / LIM141
NLD4019R / NLD401	Step 1: Plasmid pJT9R / pJT9RFR was transformed into o401RS;<br>Step 2: Plasmid pIR3DH8K digested with *Pme*I was transformed into Step 1 resulting strain to generate NLD4019R / NLD401
NLD128 / NLD128-1	Step 1: Plasmid pJT9RFR was transformed into o128R;<br>Step 2: Plasmid pIR3DH8K digested with *Pme*I was transformed into Step 1 resulting strain, named NLD128, and a superior clone was selected to generate strain NLD128-1
NLD138	Step 1: Plasmid pJT9RFR was transformed into o138R;<br>Step 2: Plasmid pIR3DH8K digested with *Pme*I was transformed into Step 1 resulting strain to generate NLD138.

Supplementary Table 6. Viability of terpene-producing yeasts in flask cultivation ($n \geq 3$). Cells sampled from flask cultivation were stained with 1.5 µM propidium iodide (PI) for 30 minutes at 30 °C in fresh MES-buffered YNB-glucose medium. Dead cells can be stained with PI and live cells exclude PI stain [6]. The PI fluorescence in single yeast cells was measured by a BD Accuri™ C6 flow cytometry with a 488 nm exciting laser and a FL3.A detector equipped with a 610 ± 10 nm bandpass filter. Per sample, 10,000 events were analysed, and viability was calculated by excluding the PI-stained population. NAA, 1-naphthaleneacetic acid.

#	Strain	Cultivation condition	Viability at 56h (%)	Viability at 72h (%)	Viability at 96h (%)	Viability at 120 h (%)
a	LIM401	2 g L^{-1} glucose; NAA added at 10h	-	78 ± 5	70 ± 2	-
b	LIM141	2 g L^{-1} glucose; without NAA	-	-	91 ± 4	-
c	LIM141	2 g L^{-1} glucose; NAA added at 10h	-	97 ± 2	84 ± 5	-
e	LIM141	2 g L^{-1} glucose; NAA added at 24h	-	-	95 ± 3	
f	NLD401	2 g L^{-1} glucose; without NAA added	87 ± 4	88 ± 0	-	-
g	NLD401	2 g L^{-1} glucose; NAA added at 0h	93 ± 1	91 ± 1	-	-
h	NLD128-1	2 g L^{-1} glucose; NAA added at 0h	-	91 ± 5	-	-
i	NLD401	2 g L^{-1} glucose; NAA added at 24h	91 ± 2	90 ± 2	-	-
j	NLD138	2 g L^{-1} glucose; without NAA	89 ± 2	86 ± 1	-	-
k	NLD138	2 g L^{-1} glucose; NAA added at 10h	88 ± 3	77 ± 3	-	-
l	NLD138	2 g L^{-1} glucose; NAA added at 24h	88 ± 2	85 ± 3	-	-
m	NLD401	2 % (v/v) ethanol; NAA added at 0h	-	-	-	77 ± 7
n	NLD138	2 % (v/v) ethanol; NAA added at 0h	-	-	-	81 ± 5

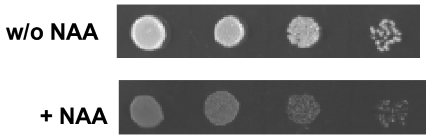

Supplementary Figure 1. Growth of the background strain o401RS on YNB 20 g L^{-1} glucose agar with or without addition of 1 mM 1-naphthaleneacetic acid (NAA). The agar plate was incubated at 30 °C for 48 hours. Replication experiment was not performed.

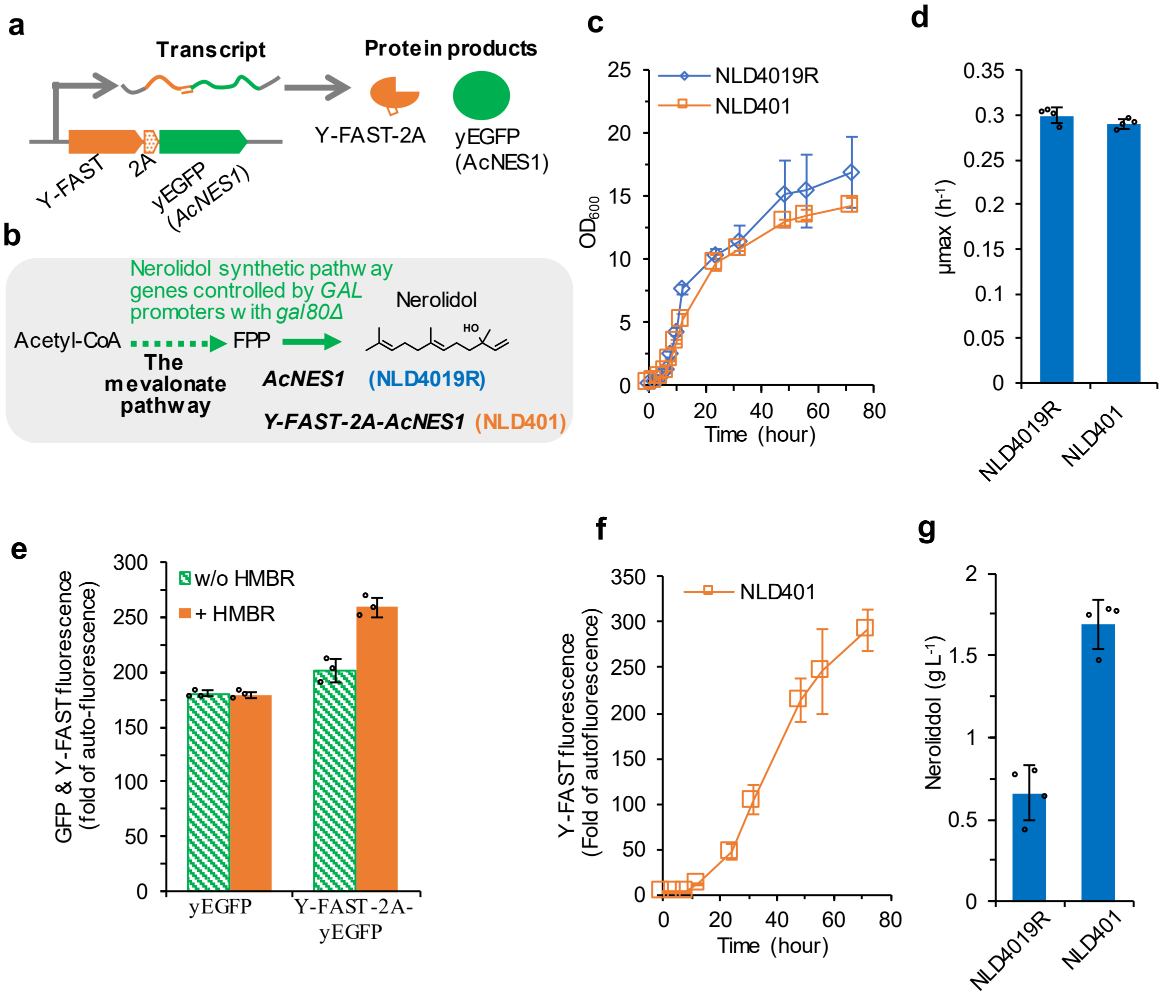

Supplementary Figure 2. Characterisation of the effects of N-terminal Y-FAST-2A tag on expression of its C-terminal fusion partner. **a** Schematic of 2A peptide-mediated polycistronic expression of *Y-FAST* and *yEGFP* or *AcNES1* (*Actinidia chinensis* trans-nerolidol synthase). **b** Schematic of engineered nerolidol synthetic pathway in strain NLD4019R (expressing AcNES1) and strain NLD401 (expressing Y-FAST-2A-AcNES1). **c, d, f, g** Characterisation of strain NLD4019R and strain NLD401. Two-phase flask cultivation with 20 g L^{-1} glucose and dodecane overlay was employed. Y-FAST fluorescence was measured in cells after incubation with 20 μM 4-hydroxy-3-methylbenzylidene rhodanine (HMBR). Y-FAST fluorescence is expressed as percentage of exponential-phase auto-fluorescence of a reference strain GH4. Mean values ± standard deviations are shown (N = 3 or 4 biological independent samples). **e** GFP and Y-FAST fluorescence in strains expressing yEGFP (strain G1H5) or Y-FAST-2A-yEGFP (strain Y2A-GFP) under the control of the *TEF1* promoter. Cells grown in exponential phase were used for the fluorescence assay. Mean values ± standard deviations are shown (N = 3 biological independent samples). Source data in **c-g** are provided as a Source Data file.

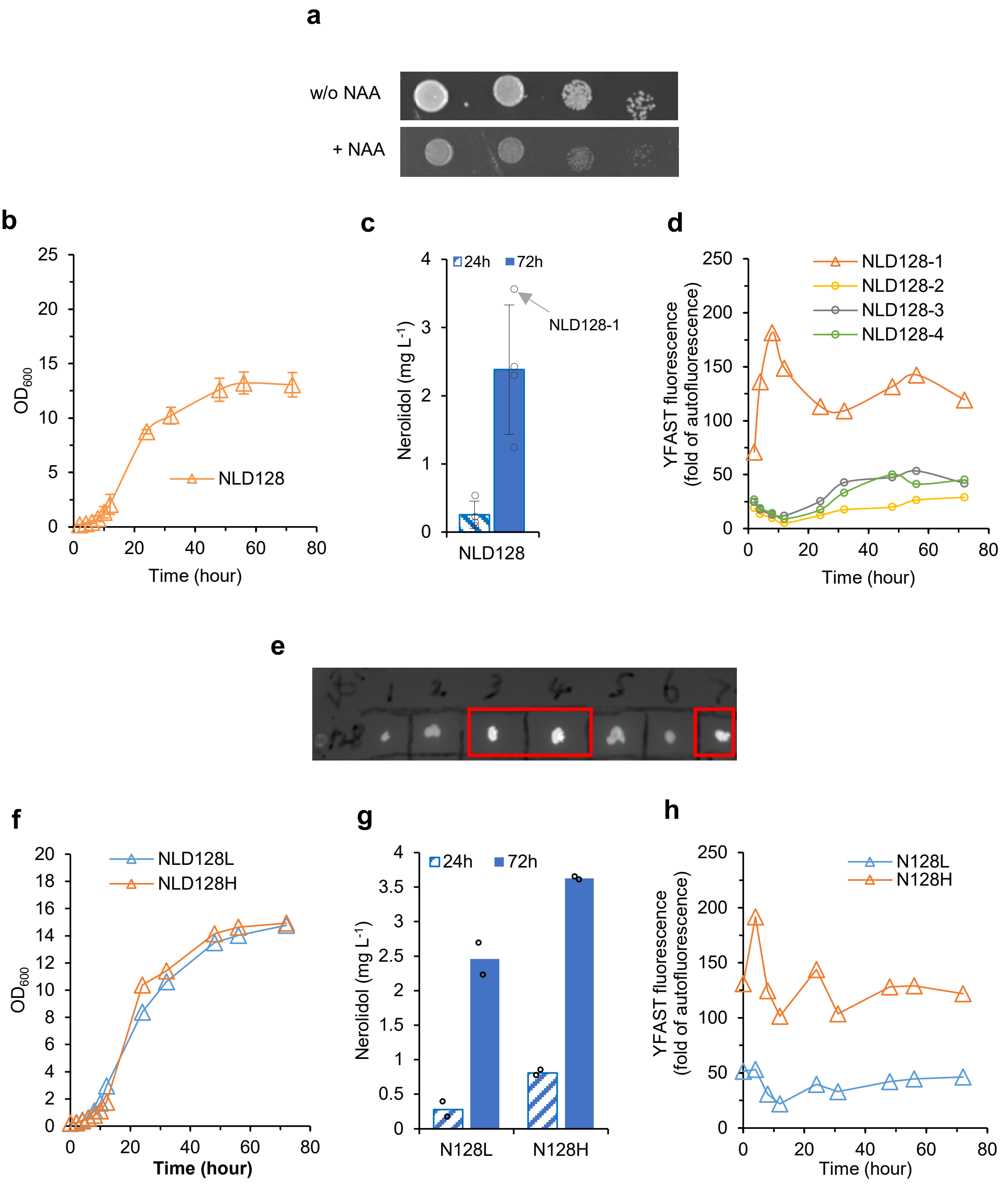

Supplementary Figure 3. Characterisation of *HXK2-AID*-CUP1* nerolidol-producing strains. **a** Growth of the background strain o128R (*HXK2-AID*-CUP1*; *GAL80* without nerolidol synthetic module) on YNB-Glucose agar with or without addition of 1 mM 1-naphthaleneacetic acid (NAA). The agar plate was incubated at 30 °C for 48 hours. **b-d** Characterisation of NLD128 (N= 4 biological independent samples: NLD128-1, NLD128-2, NLD128-3, and NLD128-4) obtained in the first batch of transformation. Mean values ± standard deviations are shown in **b** and **c** (N = 4 biological independent samples). N = 1 in **d**. **e** Examining Y-FAST fluorescence of NLD128 biological replicates obtained in the second batch of transformation. **f-h**, Characterisation of NLD128 biological replicates NLD128L (showing lower Y-FAST fluorescence in **e**) and NLD128H (showing higher Y-FAST fluorescence in **e**) obtained in the second batch of transformation. Mean values are shown in **f-g** (N = 2 biological independent samples). Source data in **c-d**, **f-h** are provided as a Source Data file.

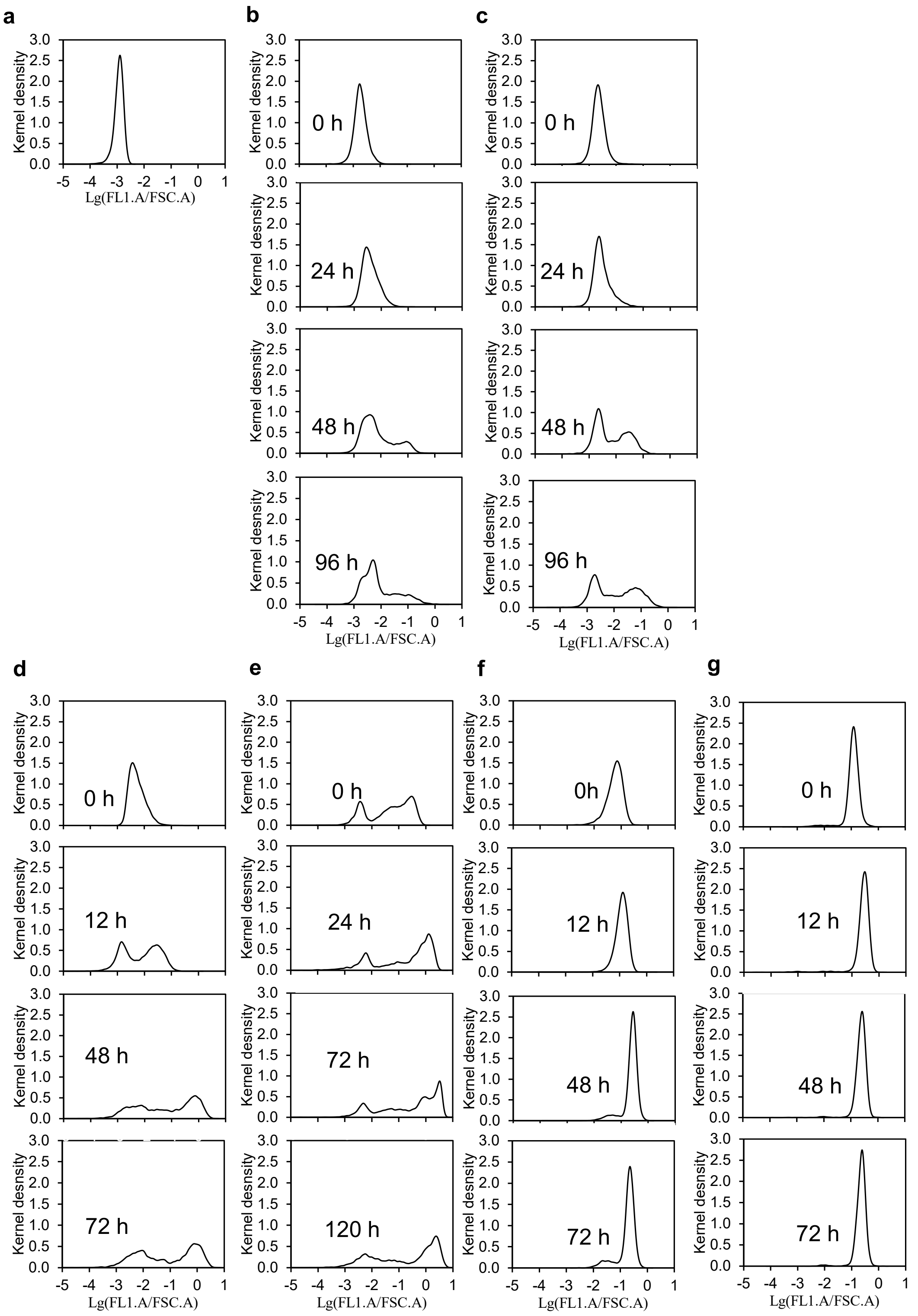

Supplementary Figure 4. Kernel density (bandwidth = 0.05) of Y-FAST fluorescence in terpene-producing strains in flask cultivations. The background fluorescence reference strain GH4 (**a**). Limonene-producing strains LIM401 (**b**) and LIM141 (**c**) with 1mM 1-naphthaleneacetic acid (NAA) added at 10 hour and 20 g L^{-1} glucose as the carbon source. Nerolidol producing strain NLD401 with NAA added at 0 hour and in pre-cultures and with 20 g L^{-1} glucose (**d**) or 2 % (v/v) ethanol (**e**) as the carbon source. Nerolidol-producing strain NLD128-1 with 20 g L^{-1} glucose as the carbon source and without NAA addition (**f**) or with NAA added at 0 hour and in pre-cultures (**g**). Kernel density was calculated using *R*, and drawn using Microsoft Excel. Data for one of biological replicates was shown. Source data are provided as a Source Data file.

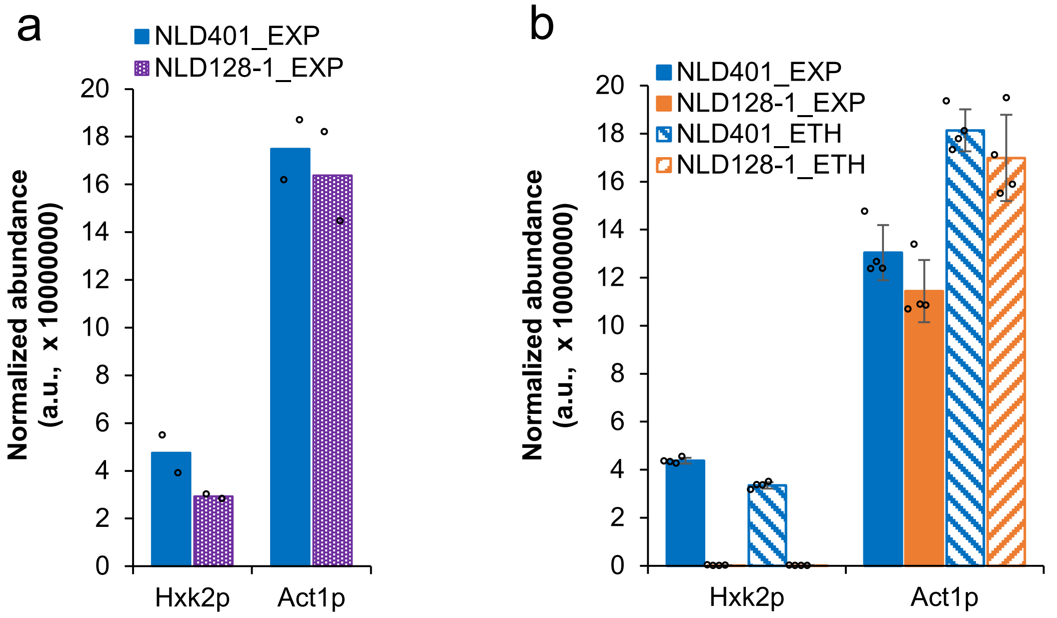

Supplementary Figure 5. Analysis of hexokinase 2 (Hxk2p) abundance in strain NLD401 (*HXK2*) and NLD128 (with auxin-inducible degradation of Hxk2p) under the conditions without addition of auxin 1-Naphthaleneacetic acid (NAA; **a**) and with addition of NAA at 0 hour and pre-cultures (**b**) through liquid chromatography mass spectrometry-based proteome assay. Yeast cells were grown through dodecane-overlayed two-phase cultivation. Cells in the exponential growth phase (EXP) were sampled when OD_{600} was in the range between 1 to 2, and cells in the ethanol growth phase (ETH) were sampled when OD_{600} was around 10 at 36 hour. The proteomics were analysed by Metabolomics Australia (Bioplatforms Australia) Queensland node through an S-Trap^{TM} sample processing protocol, Thermo Scientific LC-MS/MS equipment, and a Thermo Proteome Discoverer software for data processing (Detailed method not shown). The samples in a and b were run in separate batches through different LC-MS/MS equipment. Mean values are shown in **a** (N = 2 biological independent samples for NLD401 and independent cultures for NLD128-1). Mean values ± standard deviations in **b** (N = 4 biological independent samples for NLD401 and independent cultures for NLD128-1). Source data are provided as a Source Data file.

Supplementary results 1: A Y-FAST-2A tag

Because the expression level of the terpene synthase is important for production efficiency, we were interested in directly examining the translation from the terpene synthase transcript. We employed 2A self-cleaving peptide to enable the co-expression of *Y-FAST* and terpene synthase from a single transcript. In our design Y-FAST and an Equine rhinitis B virus-1 2A peptide, formerly characterised as the 2A peptide with the highest cleaving efficiency[7], was fused to the N-terminus of the terpene synthase, *Actinidia chinensis trans*-nerolidol synthase (*AcNES1*; Supplementary Figure 2a).

We firstly examined the effect of Y-FAST-2A fusion on the expression of its downstream gene, using yEGFP as the reporter. We fused Y-FAST-2A to the N-terminus of yEGFP and the *TEF1* promoter was used to control its transcription. The yEGFP fluorescence in yEGFP-expressing strain and Y-FAST-2A-yEGFP-expressing strain were not significantly different (Supplementary Figure 2e; two-tailed Welch's t-test $p = 0.068$) indicating no difference in yEGFP expression level. Incubating with HMBR did not change the fluorescence in yEGFP-expressing strain, as expected, whereas the fluorescence in Y-FAST-2A-yEGFP-expressing strain increased by ~22%, compared to that under the conditions without HMBR addition. The increased fluorescence is contributed by HMBR-binding Y-FAST.

Further, we used nerolidol-producing strains to test the effects of the Y-FAST-2A fusion at the N-terminus of a terpene synthase. We constructed two nerolidol producing strains, strain NLD4019R expressing nerolidol synthase gene *AcNES1*, and strain NLD401 expressing Y-FAST-2A-*AcNES1* (Supplementary Figure 2b). In both strains, anabolic genes for nerolidol synthesis were under the control of *GAL* promoters. *AcNES1* in NLD4019R and *Y-FAST-2A-AcNES1* in NLD401 were under the control of the *GAL2* promoter. Unexpectedly, the two strains showed differences in growth and nerolidol production. The *AcNES1* expressing strain NLD4019R showed slightly faster exponential growth (Supplementary Figure 2d; not significant: two-tailed Welch's t-test $p = 0.13$) and accumulated more biomass (Supplementary Figure 2c; not significant: two-tailed Welch's t-test $p = 0.165$) in the post-exponential phase than the Y-FAST-2A-AcNES1-expressing strain NLD401. Strain NLD4019R produced ~660 mg L^{-1} nerolidol at 72 hours (Supplementary Figure 2g). *Y-FAST-2A-AcNES1*-expressing strain NLD401 produced ~1.7 g L^{-1} nerolidol in the same time (Supplementary Figure 2g), an improvement by ~1.6 fold compared with strain NLD4019R. We postulated that fusing *Y-FAST-2A* to *AcNES1* N-terminus might improve its expression through uncharacterised mechanisms. As designed, we examined the induction of Y-FAST over the whole cultivation (Supplementary Figure 2f). The Y-FAST fluorescence was induced after the exponential growth phase, consistent with the regulatory pattern from the *GAL* promoter in *gal80*Δ background strain grown on glucose aerobically [4].

Supplementary result 2: Variation of Hxk2p-engineered yeast

To test the effect of Hxk2p depletion on nerolidol production, we constructed the strain NLD128 expressing TIR1 and with AID*-CUP1 fused to the C-terminal of Hxk2p. Consistent with the reference NLD401, the genes from the mevalonate pathway, farnesyl pyrophosphate synthase gene ERG20, and Y-FAST-2A-AcNES1 were controlled by GAL promoters in NLD128. We firstly characterised four NLD128 clones (Supplementary Figure 2b-d). On average, ~2.4 g L^{-1} nerolidol was produced in flask cultivation. However, a dramatic variation was observed among these four clones. The first clone, NLD128-1, produced ~3.6 g L^{-1} nerolidol in flask cultivation with NAA added at 0 hour and in pre-cultures, whereas other clones produced much less amount. The Y-FAST fluorescence levels in the low-productivity clones were much lower, compared to that in NLD128-1 and induced Y-FAST fluorescence levels in the reference strain NLD401. Further analyses are necessary to elucidate the genetic basis responsible for this variation.

To validate the repeatability of the experiments, we re-transformed the strain o128R to generate the strain NLD128 (Supplementary Table 5). Seven clones were replicated on YNB-glucose agar supplemented with HMBR. We examined the fluorescence from these seven colonies using a Safe Imager™ 2.0 Blue Light Transilluminator. We found three out of seven clones exhibited much stronger fluorescence than other clones (Supplementary Figure 2e). Two high-fluorescent clones (NLD128H) and two low-fluorescent clones (NLD128L) were characterised (Supplementary Figure 2f-h). In NLD128H, ~3.6 g L-1 nerolidol was produced at 72 hours in flask cultivation with NAA added at 0 hour and in precultures, and in NLD128 L, ~2.5 g L^{-1} nerolidol was produced. Y-FAST fluorescence in NLD128H were much higher than that in clones NLD128L. *HXK2* modifications in NLD128H and NLD128L was confirmed by DNA sequencing, and the sequence was as being designed. The causes of this variation might be due to the variations in plasmid copy number or other genetic loci.

Supplementary result 3: Induction of *GAL* promoter in terpene-producing strains

In terpene-producing strains, *GAL80* was disrupted to enable auto-induction of *GAL* promoters, which were used to regulate terpene anabolic pathways, after glucose depleted. Examining the induction of the *GAL* promoter is important for understanding the transcriptional responses to altered cultivation conditions and genetic modifications in these strains. We employed Y-FAST to examine the induction of the *GAL* promoter in flask cultivation (Figure 2d, 3f, 4d, & 4h) by analysing the mean value of Y-FAST fluorescence of populations. However, these data did not reflect the induction in individual cells. Here, we further analysed the data to understand the *GAL* promoter induction in yeast populations present in flask cultivation.

In limonene-producing strain LIM401 (without *ERG20* modified) and LIM141 (with engineered depletion of Erg20p), Y-FAST was controlled by the *GAL10* promoter. In the exponential growth phase when glucose was used as the carbon source, Y-FAST expression was repressed in both and then induced after 24 hour (Figure 2d). However, only a proportion of yeast cells showed an induced Y-FAST florescence, whereas as a proportion of cells did not (Supplementary Figure 3b-c). This indicated that the *GAL10* promoter showed a binary induction pattern in these strains in flask cultivation.

In the nerolidol-producing strains, Y-FAST and nerolidol synthase were co-expressed through a 2A-sequence-mediated bicistronic construct, *Y-FAST- 2A-AcNES1*, under the control of the *GAL2* promoter. Similar to the *GAL10* promoter, the *GAL2* promoter showed a binary induction in reference nerolidol-producing strain NLD401, when glucose was used as the carbon source (Supplementary Figure 3d). When ethanol was used as the carbon source, the *GAL* promoter is supposed to be fully derepressed. However, a binary induction of the *GAL* promoter was also observed in strain NLD401 when ethanol was used as the carbon source (Supplementary Figure 3e).

In nerolidol-producing strain NLD128 with engineered depletion of Hxk2p, the *GAL* promoter was derepressed on glucose (Figure 4g) under the conditions with or without NAA added to trigger the depletion of Hxk2p. Under the conditions without NAA addition, the *GAL* promoter was further induced after glucose depletion, whereas there was still a small proportion of cells without such induced Y-FAST levels (Supplementary Figure 3f). Under the conditions with NAA added to trigger Hxk2p depletion, yeast cells showed a homogeneous induction of Y-FAST expression. This showed that Hxk2p modification and depletion changed the induction pattern of the *GAL* promoter.

Supplementary result 4: Viability of terpene-producing strains in flask cultivation

To test whether depleting target proteins affected the strain fitness, we employed a flow cytometry-based propidium iodide exclusion assay to examine the viability of cells in flask cultivation (Supplementary Table 6).

For limonene-producing strain LIM401 (without *ERG20* modified) and strain LIM141 (with induced depletion of Erg20p), the viability at 72 hour and/or 96 hour were assayed with glucose used as the carbon source. Strain LIM141 showed the survival rates higher than that in strain LIM401 (Supplementary Table 6#a-e).

For nerolidol-producing reference strain NLD401 (without target gene modified), we examined the influence of auxin on cell viability when glucose was used as the carbon source. Compared to the strain free of auxin, adding auxin had minor effect on cell viability (Supplementary Table 6#f, #g, & #i). For the nerolidol-producing strain NLD128-1 with engineered depletion of Hxk2p, the survival rate was 91% at 72 hour (Supplementary Table 6#h), similar to that in strain NLD401 (Supplementary Table 6#g). Similarly, the nerolidol-producing strain NLD138 with engineered depletion of Acc1p did not showed a remarkable difference on cell viability (Supplementary Table 6-#j-l).

When ethanol used as the carbon source, strain NLD401 and strain NLD138 did not show a significant difference in cell viability (Supplementary Table 6#m-n).

These showed that in our cases, auxin-induced depletion of target proteins did not cause dramatic change on cell viability. However, the terpene-producing strains showed a fitness problem, as shown by a lower viability compared to farnesene-producing yeast previously reported [8].

Supplementary References

1. Peng, B., Williams, T., Henry, M., Nielsen, L. & Vickers, C. Controlling heterologous gene expression in yeast cell factories on different carbon substrates and across the diauxic shift: a comparison of yeast promoter activities. *Microbial cell factories* **14**, 91 (2015).
2. Laughery, M.F. et al. New vectors for simple and streamlined CRISPR-Cas9 genome editing in Saccharomyces cerevisiae. *Yeast (Chichester, England)* **32**, 711-720 (2015).
3. Peng, B., Nielsen, L.K., Kampranis, S.C. & Vickers, C.E. Engineered protein degradation of farnesyl pyrophosphate synthase is an effective regulatory mechanism to increase monoterpene production in Saccharomyces cerevisiae. *Metabolic engineering* (2018).
4. Peng, B., Plan, M.R., Carpenter, A., Nielsen, L.K. & Vickers, C.E. Coupling gene regulatory patterns to bioprocess conditions to optimize synthetic metabolic modules for improved sesquiterpene production in yeast. *Biotechnology for biofuels* **10**, 43 (2017).
5. Entian, K.D. & Kötter, P. in Yeast gene analysis. (eds. J.P.A. Brown & M.F. Tuite) (Academic Press, USA; 1998).
6. Deere, D. et al. Flow cytometry and cell sorting for yeast viability assessment and cell selection. *Yeast (Chichester, England)* **14**, 147-160 (1998).
7. Souza-Moreira, T.M. et al. Screening of 2A peptides for polycistronic gene expression in yeast. *FEMS yeast research* **18** (2018).
8. Meadows, A.L. et al. Rewriting yeast central carbon metabolism for industrial isoprenoid production. *Nature* **537**, 694-697 (2016).
